# *Portulaca oleracea* exhibited anti-coccidian activity, fortified the gut microbiota of Hu lambs

**DOI:** 10.1186/s13568-024-01705-4

**Published:** 2024-05-03

**Authors:** Shiheng Li, Senyang Li, Shuaiqi Liu, Shunli Lu, Jing Li, Shuqi Cheng, Sumei Zhang, Shucheng Huang, Junqiang Li, Fuchun Jian

**Affiliations:** 1 College of Animal Veterinary Medicine, Henan Agricultural University, Zhengzhou, 450046 Henan China; 2 Key Laboratory of Quality and Safety Control of Poultry Products, Ministry of Agriculture and Rural Affairs, Zhengzhou, Henan People’s Republic of China; 3 International Joint Research Laboratory for Zoonotic Diseases of Henan, Zhengzhou, 450046 Henan China

**Keywords:** Anticoccidial Activity, Gut Microbiota, Intestinal, Health *Portulaca oleracea*

## Abstract

Coccidia of the genus *Eimeria* are important pathogens that cause coccidiosis in livestock and poultry. Due to the expansion of intensive farming, coccidiosis has become more difficult to control. In addition, the continued use of anti-coccidiosis drugs has led to drug resistance and residue. Some herbs used in traditional Chinese medicine (TCM) have been shown to alleviate the clinical symptoms of coccidiosis, while enhancing immunity and growth performance (GP) of livestock and poultry. Previous in vitro and in vivo studies have reported that the TCM herb *Portulaca oleracea* exhibited anti-parasitic activities*.* In total, 36 female Hu lambs were equally divided into six treatment groups: PL (low-dose *P. oleracea*), PH (high-dose *P. oleracea*), PW (*P. oleracea* water extract), PE (*P. oleracea* ethanol extract), DIC (diclazuril), and CON (control). The treatment period was 14 days. The McMaster counting method was used to evaluate the anti-coccidiosis effects of the different treatments. Untargeted metabolomics and 16S rRNA gene sequencing were used to investigate the effects of treatment on the gut microbiota (GM) and GP. The results showed that *P. oleracea* ameliorated coccidiosis, improved GP, increased the abundances of beneficial bacteria, and maintained the composition of the GM, but failed to completely clear coccidian oocysts. The *Firmicutes* to *Bacteroides* ratio was significantly increased in the PH group. *P. oleracea* increased metabolism of tryptophan as well as some vitamins and cofactors in the GM and decreased the relative content of arginine, tryptophan, niacin, and other nutrients, thereby promoting intestinal health and enhancing GP. As an alternative to the anti-coccidiosis drug DIC, *P. oleracea* effectively inhibited growth of coccidia, maintained the composition of the GM, promoted intestinal health, and increased nutrient digestibility.

## Introduction

Coccidiosis is generally a self-limiting infection caused by coccidia of the genus *Eimeria* (Kemp LE et al. 2013). Coccidia are widely distributed globally, highly host-specific, and can infect a wide range of vertebrates, including cattle, sheep, pigs, rabbits, chickens, and ducks (Yun CH et al. 2000; Keeton STN et al. 2018). Massive parasitic reproduction of coccidia causes severe damage to the mucosa of the host digestive tract, which reduces digestion and absorption of nutrients, resulting in diarrhea, stunted growth, and even death (Barba E et al. 2022). The global economic cost of coccidiosis control is estimated at around 13 billion USD annually (Blake DP et al. 2020). With the change to animal husbandry from free-range to large-scale intensive farming, the stocking density continues to increase, which leads to rapid spread of coccidia. Besides, it is difficult to prevent and control coccidiosis because of the high reproductive potential of ubiquitous coccidian oocysts (Allen PC et al. 2002). At present, antibiotics and triazines are commonly used for treatment of coccidiosis. However, the use of chemical-based drugs in animal husbandry is no longer sufficient due to widespread drug resistance, drug residues in food, and threats to the environment (Daugschies A et al. 1998; Waller PJ 2006; Paul SM et al. 2010; Bacanlı M. Başaran N. 2019). And the cost of drug development continues to rise, finding alternative anti-coccidian drugs has become an urgent priority.

Various natural plant products used in traditional Chinese medicine (TCM) are effective for treatment of parasitic diseases. Herbs used in TCM for control of coccidiosis have the advantages of wide availability, broad-spectrum applications, safety, environmental compliancy, and economic efficiency. The antimalarial drug artemisinin is reported to significantly alter wall formation of coccidian oocysts, resulting in impaired oocyst development and reduced sporulation rates in chickens (del Cacho E et al. 2010). Moreover, the combination of the plant extract berberine and amprolium was shown to significantly decrease the oocysts per gram (OPG) values of coccidia as compared to amprolium alone. In addition, the combination of berberine and amprolium was found to improve growth performance (GP) (Malik TA et al. 2016). The active ingredients of herbs used in TCM primarily include flavonoids, biophenols, alkaloids, terpenoids, and polyphenols. However, various extraction processes are required to fully activate and enhance the efficacy of these active compounds. And the roots, stems, and leaves of some plants used in TCM are toxic and must be processed before use. Some medicinal plants used in TCM are toxic in relatively large dosages, but have few side effects that after appropriate processing. Hence, the uncertainty in the dosage and form of TCM is challenging for treatment of coccidiosis.

*Portulaca oleracea* is an annual succulent in the family Portulacaceae used in various medicines and cosmetics (Juraimi AS et al. 2009; Uddin K et al. 2017). The stems and leaves of *P. oleracea* have anti-inflammatory and analgesic effects, and are used in TCM for treatment of gastrointestinal and liver diseases (Liu L et al. 2000). *P*. *oleracea* has also been used to prevent and treat gastrointestinal parasitic diseases in sheep (Cai Y et al. 2004). However, most previous studies have been conducted using avian or non-ruminant species or only in comparison to chemical deworming agents. Various in vivo and in vitro studies have shown that the gut microbiota (GM) and related metabolites play important roles in resistance to pathogens. However, relatively few studies have investigated the use of *P. oleracea* to modulate the composition of the GM and metabolite levels to influence the GP and intestinal health of ruminants.

Coccidia infection alters the composition of the GM both directly and indirectly by inducing changes to physiological characteristics, permeability, and antimicrobial peptide production (Zaiss MM, Harris NL. 2016).The pathophysiology of coccidia mainly includes damage to the intestinal mucosa, immunological inflammation, and changes to the GM (Lu C et al. 2021). The GM is a dynamic population of microorganisms that maintains a symbiotic relationship with the gut mucosa (Sebastián Domingo JJ. 2018). Changes to the structural composition of the GM, particularly the ratio of *Firmicutes* to *Bacteroidetes* (F/B ratio), can impact colonization of parasites in the host, infection status, and management of parasitic diseases (White EC et al. 2018; Wen JJ et al. 2021). Besides, some metabolites produced by bacteria in the host gut are relevant to the intestinal barrier. Alterations to the microbial metabolites tryptophan, indoleacetic acid, and indole acetaldehyde regulate intestinal barrier function via the aryl hydrocarbon receptor (Lamas B et al. 2016). However, further investigations are needed to elucidate the mechanism of action employed by *P. oleracea* to alter the composition of the GM and related metabolites during coccidia infection.

In the study, the impact of low and high doses of *P. oleracea*, as well as water and ethanol extracts, on the number of coccidia in lambs were investigated. Additionally, the impacts of *P*. *oleracea* on the composition of the GM and related metabolites during coccidia infection were analyzed. The results showed that *P*. *oleracea* decreased the OPG values of coccidia among the different treatment groups, but not as significantly the anti-coccidian drug diclazuril (DIC) in the early stages of treatment. However, the duration of efficacy was relatively longer for *P. oleracea*, which resulted in lower OPG values. The use of *P. oleracea* improved the mean daily weight gain (DWG), diversity of the GM, and F/B ratio of sheep. Overall, the findings of this study support dietary supplementation with *P. oleracea* as a substitute for anti-coccidian drugs and antibiotics.

## Methods

### Study approval

**Fig. 1:**
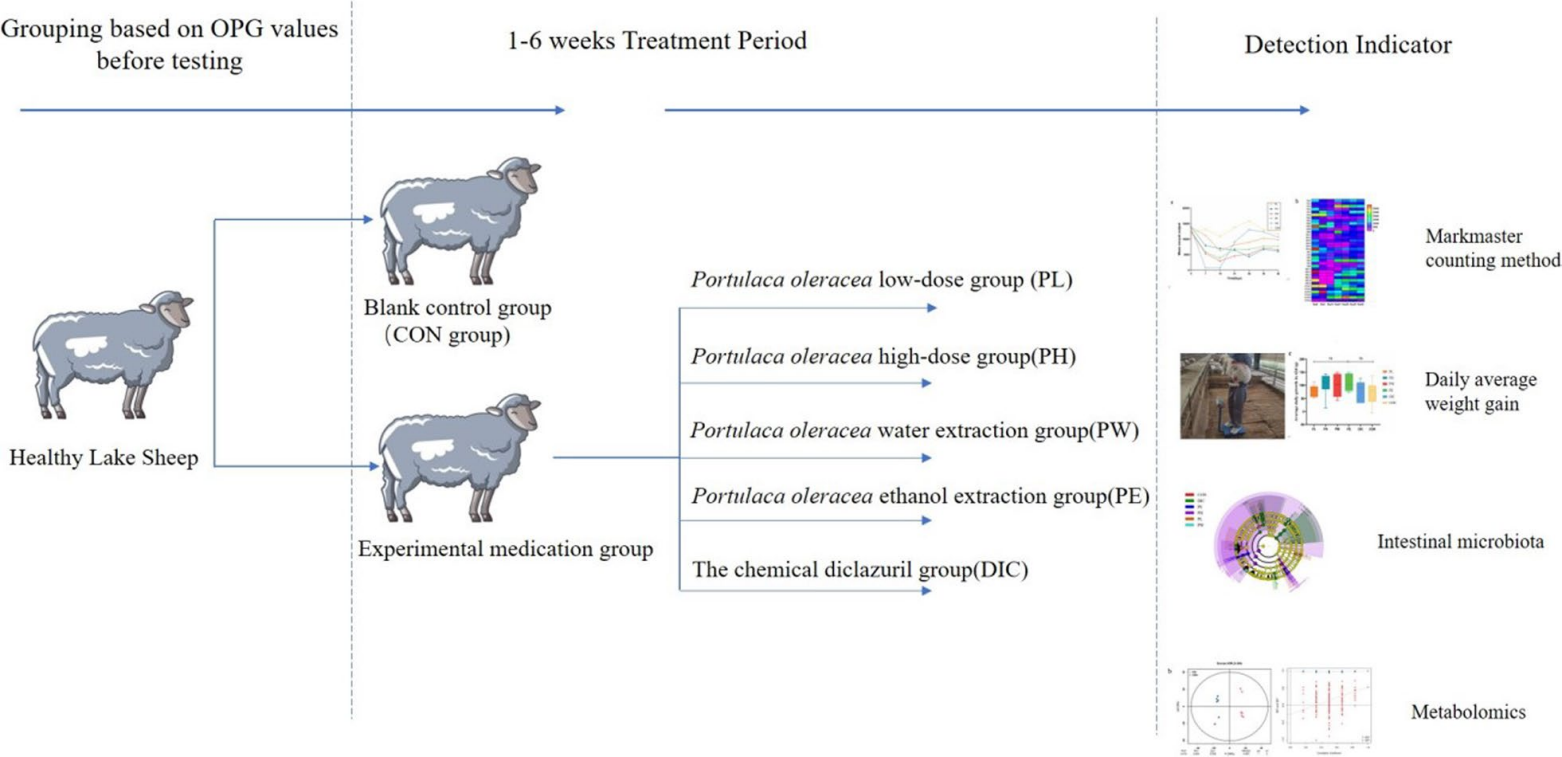
A flowchart of the experiment. In total, 36 female Hu lambs (age, 1.5 months; mean body weight [BW], 13.08 ± 0.31 kg) were divided into 6 groups of 6 animals each. Lambs in the CON group were fed a basal diet. Lambs in the DIC group were fed the basal diet and orally administered DIC at 1 mg/kg BW for 2 consecutive days. Lambs in the PL group and PH group were fed the basal diet supplemented with *P. oleracea* at 30 g/day and 120 g/day for 14 days. Lambs in the PW group and PE group were fed the basal diet and orally administered a water extract and ethanol extrac of *P. oleracea* respectively at 30 mL/day for 14 days. Lambs in thewere fed the basal diet and orally administered ant of *P. oleracea* at 30 mL/day for 14 days

The study protocol was approved by the Institutional Animal Care and Use Committee of the College of Animal Medicine, Henan Agricultural University (approval no. 17–0126), and conducted in accordance with the Guide for the Care and Use of Agricultural Animals in Research and Teaching. A flowchart of the experimental design is provided in Fig. 1.

### Materials and reagents

*P. oleracea* was purchased from Yuzhou Qianhe Pharmaceutical Co., Ltd. (Yuzhou, China) Originating in Hubei, China, the picking season is autumn. DIC premix (0.5%) was obtained from Hefei Zhonglong Shenli Animal Pharmaceutical Co., Ltd. (Hefei, China). Food-grade ethanol (75%) was acquired from Shandong Xintai Huayuan Medical and Sanitary Products Co., Ltd. (Xintai, China). A McMaster counting chamber to quantify coccidian oocysts was procured from Henan Agricultural University (Zhengzhou, China).

### Preparation of herbal extracts and composition determination

The herbal extract was prepared by solvent extraction. In brief, the herbs were crushed, macerated for 6 days in a mixture of water and 70% ethanol (1:10), and filtered several times through four layers of gauze and a 60 mesh until there was no residue. Then, the filtrate was evaporated in a boiling water bath and the resulting paste was fixed in saline. The final concentration of the extract was 1 g/1 mL of solvent.

Based on the UHPLC-QE Orbitrap platform, metabolomics qualitative and quantitative analysis of ethanol extract samples from traditional Chinese medicine.

### Animals and experimental design

In total, 36 female Hu lambs (age, 1.5 months; mean body weight [BW], 13.08 ± 0.31 kg) were obtained from a large-scale sheep farm and divided into six groups of six animals each. Fecal samples were collected two times per day for 3 days before the experiment and coccidia OPG values were calculated. Lambs in the blank control (CON) group were fed a basal diet. Lambs in the DIC group were fed the basal diet and orally administered DIC at 1 mg/kg BW for 2 consecutive days. Lambs in the low-dose *P. oleracea* (PL) group and high-dose *P. oleracea* (PH) group were fed the basal diet supplemented with *P*. *oleracea* at 30 and 120 g/day respectively for 14 days. Lambs in the *P. oleracea* water extraction (PW) group and ethanol extraction (PE) group were fed the basal diet and orally administered a water and ethanol extract of *P*. *oleracea* respectively at 30 mL/day for 14 days. Feed was offered in equal amounts at 07:00 and 18:00 h daily. All animals had ad libitum access to drinking water. The study period was 42 days.

### Sample collection

Fecal samples were collected and OPG values were calculated on days 0, 7, 14, 21, 28, 35, and 42. Fecal samples collected on day 14 were stored at − 80 °C for genomic sequencing of the GM. The BW of each lamb was measured in the morning on days 0, 14, and 42.

### Calculation of coccidia OPG

The OPG value was calculated using the McMaster method, The McMaster egg counting method is a frequently utilized technique in parasitology research. The procedure includes placing 2 g of fresh feces in a clean 100 mL beaker. Initially, 8 mL of saturated salt solution is added, followed by crushing and mixing, and then adding 50 mL of saturated salt solution. After thorough mixing, the mixture is promptly filtered using gauze or a 60 mesh sieve. Subsequently, the filtrate is transferred to two counting chambers. The sample is placed on a microscope stage for 3–5 min and examined under a 10 × objective lens. Each counting room contains 100 squares with a volume of 0.15 ml (1 × 1 × 0.15 cm). By counting the number of oocysts n1 and n2 in each of the 100 squares in two counting rooms, the OPG value (number of oocysts per gram of feces) is calculated using the formula OPG = [(n1 + n2)/(2 × 0.15)] × 60 ÷ 2. Here, (n1 + n2)/2 represents the average number of oocysts per counting chamber, 0.15 denotes the effective volume of each counting chamber (0.15 mL), 60 is the total volume of fecal fluid (60 mL), and 2 is the amount of feces used (2 g).

### DNA extraction and 16S rRNA sequencing

DNA was extracted from the fecal samples using the FastDNA^™^ SPIN Kit for Soil (MP Biomedicals, Heidelberg, Germany) as described in the manufacturer’s instructions but with slight modifications. Briefly, 250 mg of each fecal sample were homogenized in the buffer provided with the kit using a FastPrep-24^™^ Instrument (MP Biomedicals). Cell lysates were separated by centrifugation (14,000 × *g*, 15 min) and proteins were precipitated from the supernatant. DNA was diluted with pre-warmed (55 °C) purified water and quantified using a NanoDrop^™^ spectrophotometer (Thermo Fisher Scientific, Waltham, MA, USA).

Amplify and sequence the V1-V2 region of the 16S rRNA gene for each sample according to previous research methods (Camarinha-Silva A et al. 2014). The primer pair 27F and 338R was used to amplify the target region, with a slightly modified sequence of the primer 27F (AGRGTTHGATYMTGGCTCAG). The quality of the obtained amplicons was assessed by agarose gel electrophoresis. The qualified amplicons were sequenced with an Illumina MiSeq^™^ instrument (Illumina, Inc., San Diego, CA, USA) in paired-end mode (2 × 250 base pairs). Sequence reads were quality filtered and assembled using mothur software (Schloss PD et al. 2009). Reads with an mean quality score < 20, total length > 355 base pairs (bp), any primer or barcode mismatch, and homopolymer stretches with > 8 Oran characters were excluded. The reads were checked for the presence of chimeras and clustered into operational taxonomical units (OTUs) at 97% identity (Wang Q et al. 2007). OTUs appearing only once across all samples or with fewer than 10 reads were manually deleted. The remaining OTUs were finally assigned to the closest taxonomical representative using the Sequence Match tool (Lighten J et al. 2014).

The abundance of each OTU was assessed using Primer 6 v.6 software (PRIMER-E Ltd., Plymouth, UK). Statistical differences across time points among the treatment groups were identified using analysis of variance (ANOVA) with permutations.

### Metabolomic profiling of fecal samples

Fecal samples (50 mg) were homogenized in extraction buffer (1000 μL; methanol, acetonitrile, and water at 2:2:1 chilled to − 20 ℃; and 20 μL of an internal standard) using a ball mill for 4 min at 45 Hz, ultrasonicated at 5 min, and incubated for 1 h at − 20 ℃ to precipitate the proteins. Following centrifugation for 15 min at 12000 rpm and 4 ℃, 20 μL aliquots of the supernatants were used for liquid chromatography–mass spectrometry.

Follow-up analysis was performed after normalizing the original peak area to the total peak area. Principal component analysis and Spearman correlation analysis were performed to assess the repeatability of the samples within groups and the quality of the samples. Classification and associated pathways of the identified compounds were determined in reference to the Kyoto Encyclopedia of Genes and Genomes (KEGG; https://www.genome.jp/kegg/), Human Metabolome Database (https://hmdb.ca/), and Lipid Maps database (https://www.lipidmaps.org/). The variable importance in projection (VIP) score was calculated by Orthogonal Projections to Latent Structures Discriminant Analysis (OPLS-DA) using the multiple cross-validation method. The screening criteria for differential metabolites were fold change (FC) > 1, probability (*p*) value < 0.05, and VIP score > 1. The significance of differential metabolites associated with KEGG pathways was calculated using the hypergeometric distribution test.

### Analytical procedures and statistical analysis

The efficacy of *P*. *oleracea* and extracts was assessed based on survival rates, changes to OPG values, DWG, and other indicators. Pooled oocyst counts for each group were performed in accordance with the McMaster counting method. Changes to OPG values were assessed using one-way ANOVA. The average DWG was calculated as (mean final weight of live lambs in the pen) − (mean initial weight of all lambs in the pen)/number of days. The data are presented as the mean ± standard deviation. Statistical analyses were conducted with IBM SPSS Statistics for Windows, version 19.0. (IBM Corporation, Armonk, NY, USA). Figures were generated using Prism 7 software (GraphPad Software, Inc., San Diego, CA, USA). The significance of differences among the six groups was determined by one-way ANOVA. The least significant difference test was used for data exhibiting homogeneity of variance; otherwise, Tamhane’s T2 analysis was selected. The Pearson’s correlation coefficient was determined to identify linear correlations. A *p* value ≤ 0.05 was considered statistically significant. A *p* value ≤ 0.01 is considered highly statistically significant.

## Results

### Chemical composition analysis of P. oleracea extract

Using UHPLC-QE Orbitrap platform to annotate *P. oleracea* water and ethanol extracts, a total of 2831 metabolites were annotated. The components of the *P. oleracea* ethanol extraction group (PE) extract are Nicotinurate, Pantothenic Acid, and Genistein, L-Homocysteine, Furylacetone, Biotin, 3,4-Dihydroxyphthalate, etc. Genistein, Biotin, Engeletin, and Ephedrine are common active ingredients in plants, purslane alcohol extracts are mainly plant active ingredients and some growth promoting substances. The main components of purslane water extract (PW) are Thymine, Sorbic acid, L-Homocysteine, Nicotinurate, Biotin, Methyloscitric acid, Rotenone, etc. Sorbic acid, Rotenone, Engeletin are active ingredients in plants. The detailed results of ingredient content are shown in Table 1

**Table 1:** Content of water and ethanol extracts from *P. oleracea*

PE compounds	Relative abundance	PW compounds	Relative abundance
Nicotinurate	3008.225387	Thymine	17335.64018
Pantothenic Acid	2783.052828	Sorbic acid	1956.524079
Genistein	871.5764289	L-Homocysteine	583.0955917
L-Homocysteine	728.3894644	Nicotinurate	528.8977342
Furylacetone	633.7749364	Furylacetone	406.9841946
Biotin	602.4740584	Biotin	378.4208285
3,4-Dihydroxyphthalate	407.773725	serinamide	191.0706502
p-aminobenzoyl glutamate	243.622553	Methylisocitric acid	91.45481262
serinamide	218.5708458	Rotenone	67.41083481
Isoleucyl-Cysteine	202.7208139	p-aminobenzoyl glutamate	40.57738239
Engeletin	151.1668341	Premithramycin A3'	39.61825029
Ephedrine	70.06475459	Engeletin	22.19761833

### Effect of P. oleracea on excretion of coccidian oocysts

**Fig. 2:**
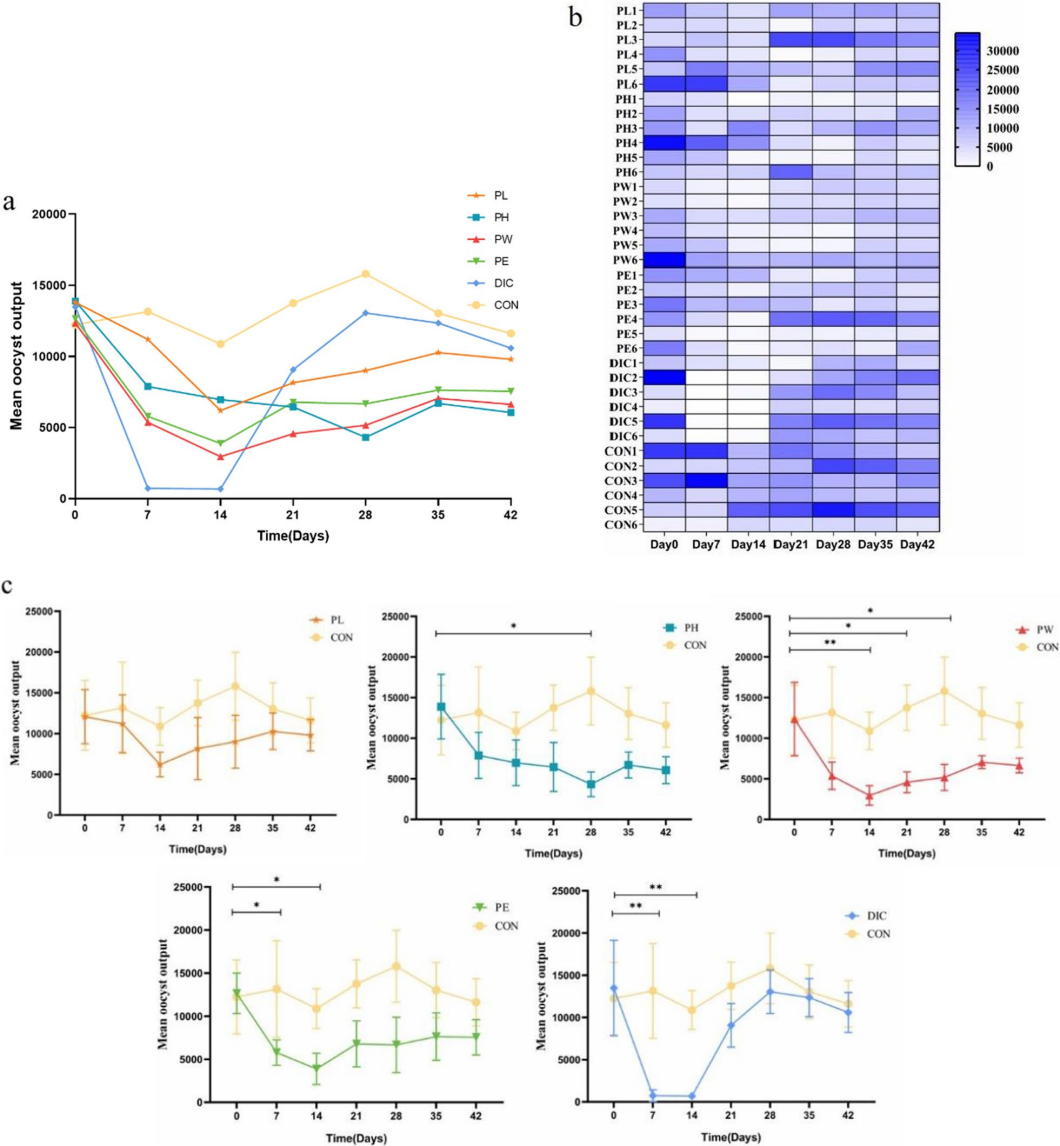
Effects of different treatments on coccidia in lambs. **a** line chart of mean OPG values of the different treatment groups. **b** Heat map of changes to OPG values, where higher OPG values are indicated by brighter colors. **c** Line graph of changes to OPG values. Analysis of variance was used to compare the OPG values. Asterisks indicate significant differences (*0.01 < *p* ≤ 0.05; **0.001 < *p* ≤ 0.01; ***p ≤ 0.001)

As shown in Table 2, the OPG values of the PL, PH, PW, PE, and DIC groups on day 7 were 11200, 7883, 5367, 5783, and 733, respectively. The OPG values were significantly lower in the PW and DIC groups than the CON group (*p* ≤ 0.05 and 0.01, respectively). On day 14, the OPG values were significantly lower in the PW and DIC groups than the CON group (*p* ≤ 0.01). On the final day of treatment (day 28), the OPG values were significantly lower in the PH, PW, and PE groups than the CON group (*p* ≤ 0.05), while there was no significant difference between the DIC and CON groups (*p* > 0.05). The OPG values of each *P. oleracea* treatment group at specific time points are shown in the line graph presented in Fig. 2a. The OPG values of the different *P*. *oleracea* treatment groups had decreased as compared to the initial values, with the most obvious decrease in the DIC group. After 14 days, the OPG value of the DIC group suddenly increased, while the OPG values of the other drug groups were relatively stable. DIC had the strongest anti-coccidian effect, but efficacy was only maintained for 14 days, followed by the PW group. This finding suggests that DIC has a relatively short-term anti-coccidian effect. A heat map of the OPG values over time is presented in Fig. 2b. After medication, the color of the lambs gradually turned white, with the highest proportion and degree observed in the DIC group. Some of the sheep appeared blue on day 14. However, the heat map of the different treatment groups indicates a trend of turning white. Changes to the OPG values of individual sheep collectively reflect the overall change to the group.

**Table 2:** Coccidia oocyst excretion of different treatment groups of *P*.*oleracea* (a, b, c values with different superscripts differ significantly (*P* ≤ 0.05)

Group	Mean OPG before medication	Mean OPG after medication					
		7 d	14 d	21 d	28 d	35d	42d
PL	13800 ± 7200^a^	11200 ± 8679^ab^	6200 ± 3704^ab^	8150 ± 9328^ab^	9000 ± 7934^ab^	10,267 ± 5493^a^	9800 ± 4726^a^
PH	13883 ± 9757^a^	7883 ± 6940^ab^	6967 ± 6869^ab^	6450 ± 7378^ab^	4317 ± 3719^b^	6700 ± 3899^a^	6067 ± 4046^a^
PW	12350 ± 11068^a^	5367 ± 4100^b^	2950 ± 2944^B^	4567 ± 3135^b^	5167 ± 3925^b^	7050 ± 1938^a^	6633 ± 2220^a^
PE	12650 ± 5747^a^	5783 ± 3624^ab^	3883 ± 4447^b^	6783 ± 6532^ab^	6667 ± 7872^b^	7633 ± 6746^a^	7550 ± 5014^a^
DIC	13483 ± 8720^a^	733 ± 1700^B^	683 ± 1140^B^	9067 ± 6323^ab^	13050 ± 8206^ab^	12350 ± 5532^a^	10583 ± 5795^a^
CON	12233 ± 10504^a^	13150 ± 5616^a^	10883 ± 5648^a^	13750 ± 6843^a^	15800 ± 10226^a^	13033 ± 7816^a^	11616 ± 6728^a^

Treatment with *P. oleracea* and the extracts tended to decrease the OPG values. However, anti-coccidian effect was weaker in the PL group than the PH, PW, and PE groups. There were significant differences in OPG values between the PW and CON group on days 7, 14, and 21, with the lowest OPG value on the 42th day of the experiment (*p* ≤ 0.05). There were also significant differences in OPG values between the PE and CON groups on days 7 and 14, and between the PH and CON groups on the 28th day of the experiment (*p* ≤ 0.05). These results show the *P. oleracea* extracts had better anti-coccidian effects, which may be due to greater release of effective components of *P. oleracea* after extraction. Although DIC initially exhibited strong anti-coccidian effects on the 7th and 14th day of the experiment (*p* ≤ 0.01), the OPG value immediately increased with no difference as compared to the CON group. Notably, the coccidia were not completely eradicated in the PH, PW, and PE groups, but infection was ameliorated for prolonged periods.

### Effect of P. oleracea on GP

**Fig. 3:**
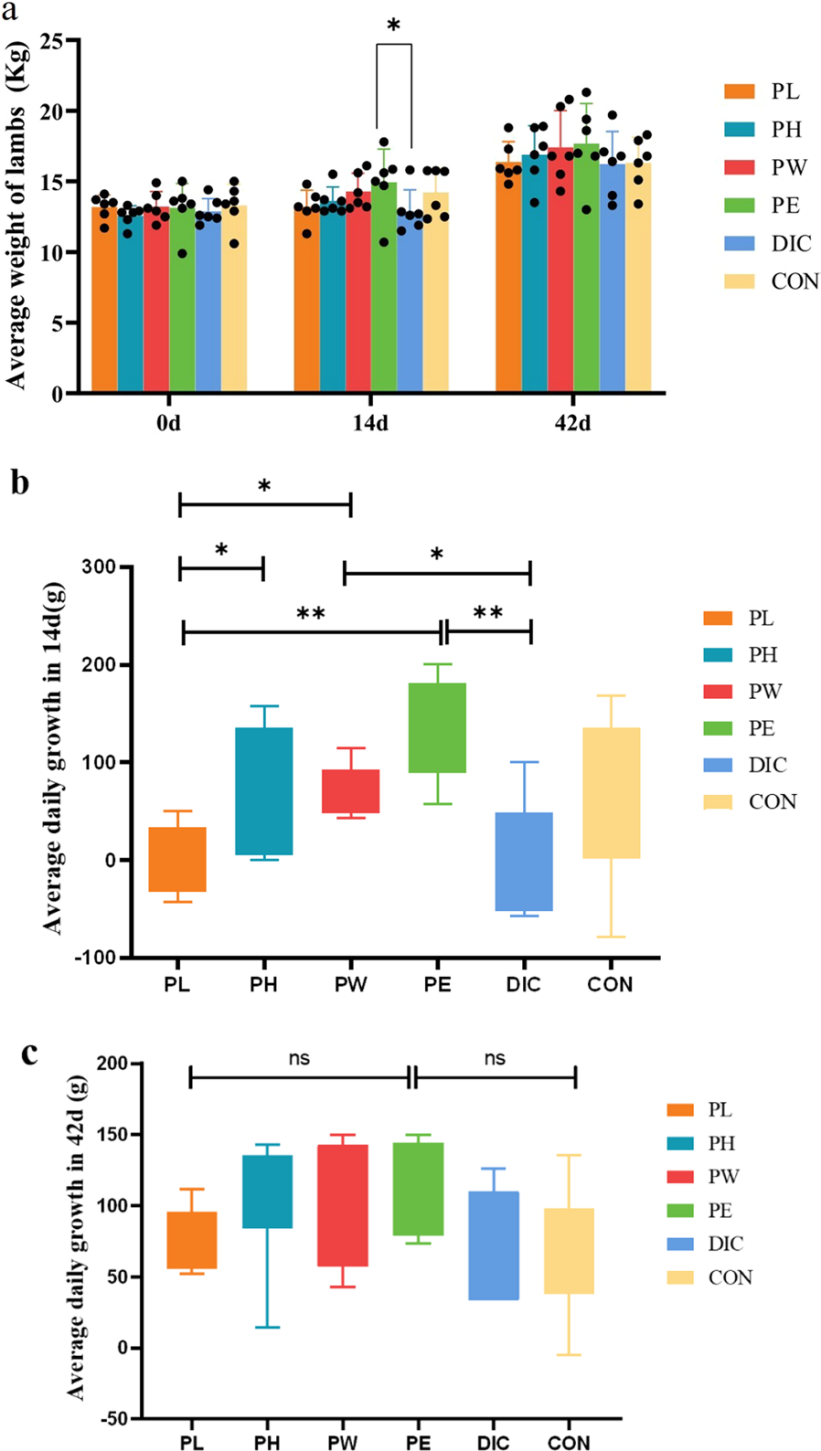
Effect of different treatments on GP of sheep. **a** Mean weight of sheep in each group at different times. **b** Mean DWG in each group during the dosing period (14 days). **c** Mean DWG of each group throughout the trial period (42 days). (*0.01 < *p* ≤ 0.05; **0.001 < *p* ≤ 0.01; ***p ≤ 0.001)

As shown in Fig. 3a, the mean BW of lambs on days 14 and 42 was higher in each *P. oleracea* treatment group as compared to the CON group. On day 14, the mean BW was significantly higher in the PE group than the DIC group (*p* ≤ 0.05). As shown in Fig. 3b, the mean DWG during the 14 day treatment period was significantly higher in the PH and PW groups than the PL group (*p* ≤ 0.05), and significantly higher in the PE group than the PL and DIC groups (*p* ≤ 0.01). As shown in Fig. 3c, the mean DWG was higher in each treatment group than the CON group throughout the entire 42 day experimental period. The mean DWG was highest in the PH, PW, and PE groups, but there was no significant difference as compared to the CON group (*p* > 0.05). Collectively, these results demonstrate that supplementation with *P. oleracea* can improve the GP of sheep and a single dose of DIC has relatively limited effects.

### P. oleracea is beneficial to maintain the composition of the GM

**Fig. 4:**
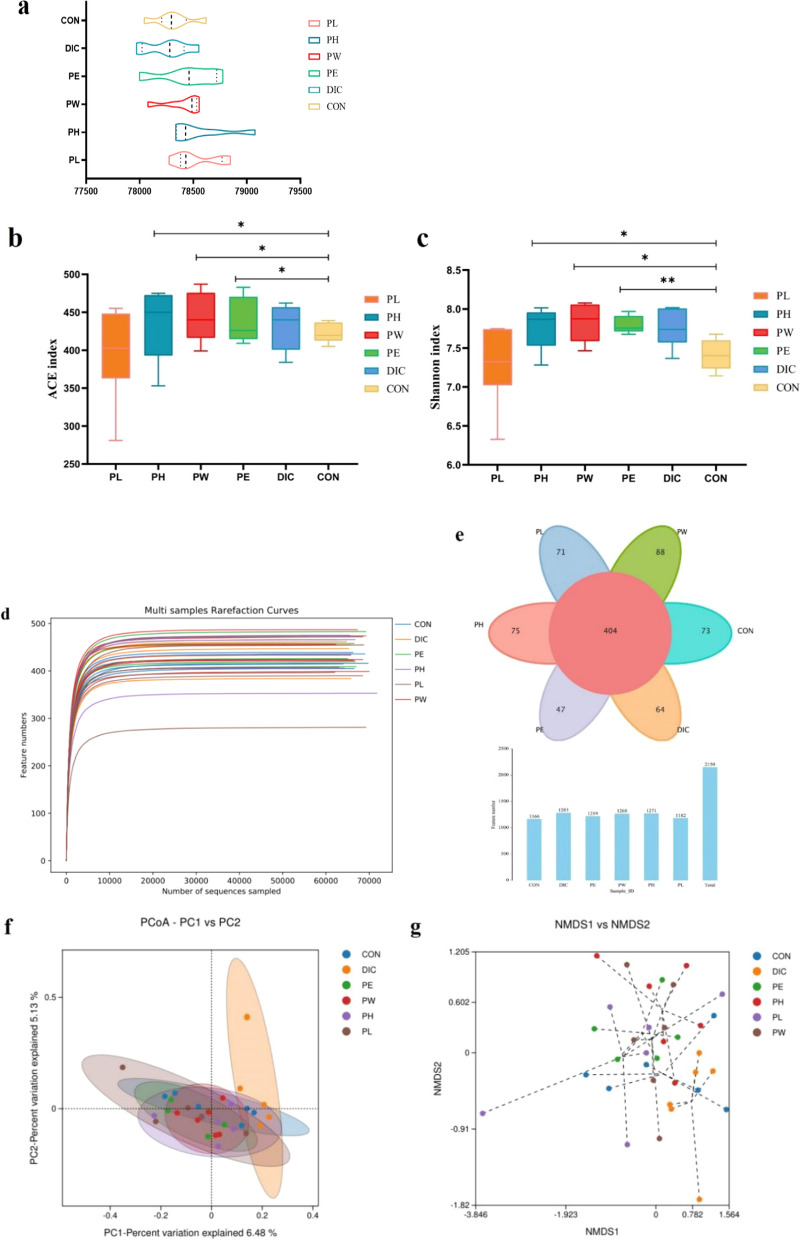
Effect of *P*. *oleracea* on the overall structure of the GM. **a** Sample coverage of the six treatment groups. **b** Chao1 indices of the six treatment groups. **c** Shannon indices of the six treatment groups. **d** Dilution curves to compare the richness of species among samples and to assess the size of the cohort. **e** Venn diagrams of the OTUs of each group. **f** PCoA plots based on Spearman distances are colored by time point. The significance of differences among the groups was determined by analysis of similarity. **g** NMDS among groups was conducted by one-way ANOVA (*p < 0.05; **p < 0.01)

The roles of the GM in health and disease are well established. In the present study, 16S rRNA analysis was conducted to clarify the effects of dietary additives on the composition of the GM. The term “clean reads” refers to high-quality reads obtained after quality control of the original sequence (Fig. 4a). As shown in Fig. 4b, the richness of the GM (ACE index) was significantly higher in the PH, PW, and PE groups than the CON group (*p* ≤ 0.05), while there was no difference in the PL and DIC groups as compared to the CON group. The diversity of the GM (Shannon index) was significantly higher in the PH and PW groups than the CON group (*p* ≤ 0.05; Fig. 4b; Fig. 4c), and even more pronounced in the PE group than the CON group (*p* ≤ 0.01), while there was no significant difference between the PL and CON groups. These results indicate that the high-dose of *P. oleracea* as well as the water and ethanol extracts increased the diversity of the GM, while the low-dose of *P*. *oleracea* also improved diversity, but to a lesser extent. A dilution curve is generally used to reflect the reliability of the diversity results of sequencing data (Amato KR, et al. 2013). As shown in Fig. 4d, the dilution curve gradually flattened, indicating that the diversity results are reliable.

Clustering OTUs at 97% similarity reflects differences in sequencing data. As shown in Fig. 4e, the number of OTUs was lower in the CON group than the PH and PW groups, demonstrating that *P*. *oleracea* influenced the composition of the GM. In order to further study the similarities and differences in the composition of the GM among the groups, non-metric multidimensional scaling (NMDS) and principal co-ordinate analysis (PCoA) were performed. Cluster analysis uses a tree structure to describe and compare similarities among multiple samples. As demonstrated by the results of NMDS (Fig. 4f) and PCoA (Fig. 4g), the CON group is similar in distance to the PH and PE groups demonstrate that the colony structure of the lambs, when treated with *P. oleracea*, is similar to the colony composition of normal lambs. The large distance between the DIC group and other groups indicates that the use of DIC has changed the colony composition of GM. The effects of different properties of chemical drugs and traditional Chinese medicine on GM may vary.

### Differences in dominant microbial communities among the treatment groups

**Fig. 5:**
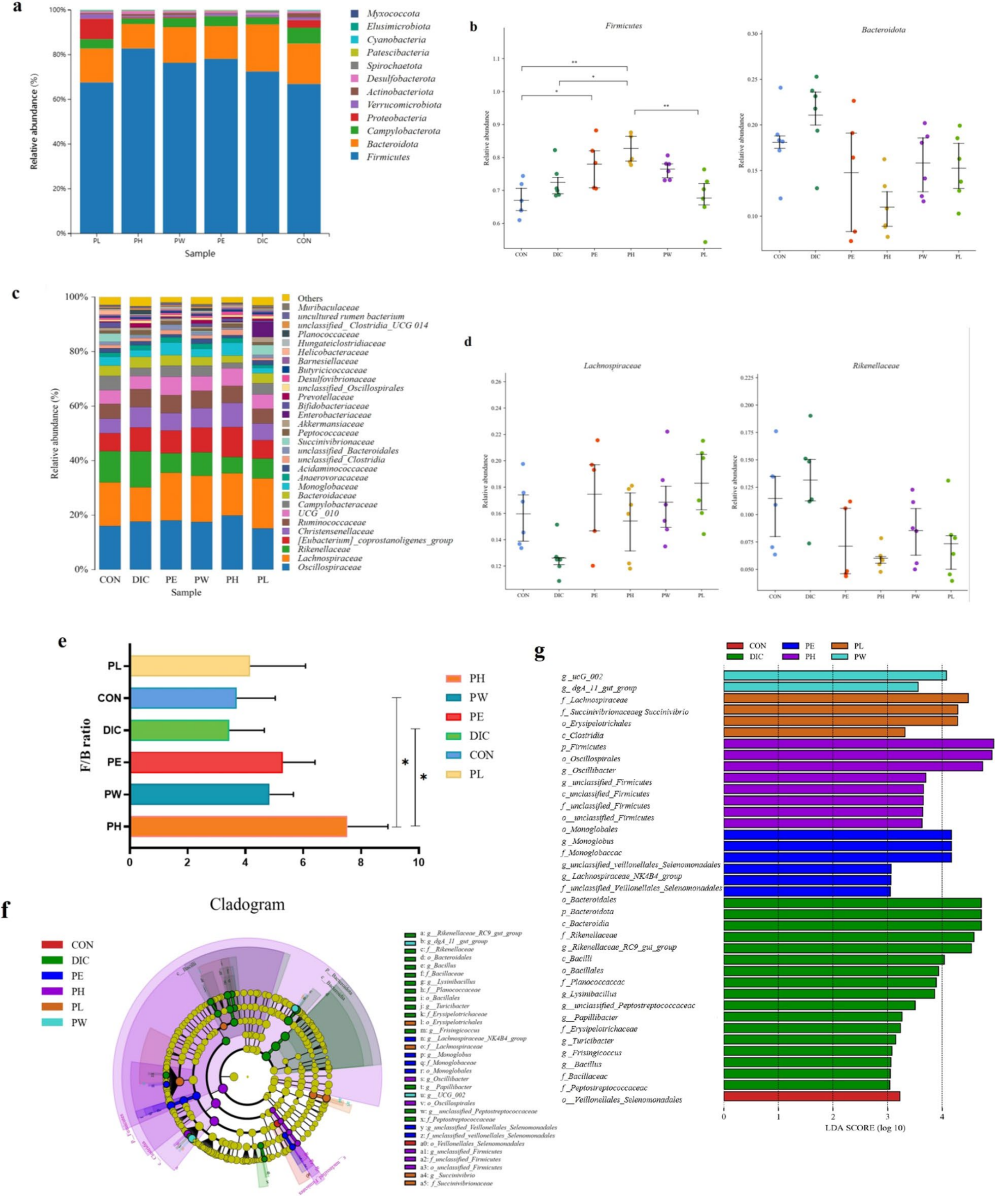
The dominant members of the GM at the phylum and genus levels. **a** Composition of the GM (top 8 phyla) of each group. **b** Relative abundance of *Firmicutes* and *Bacteroides* of the six groups. **c** Relative abundances of the top 30 genera of each group. **d** Relative abundances of *Lachnospiraceae* and *Rikenellacae* of the six groups/ **e** F/B ratio. **f** and **g** Taxonomic cladogram obtained by LEfSe of the six groups. Biomarker taxa are highlighted with colored circles and shaded areas. The diameter of each circle reflects the abundance of the taxa. **b**–**d**, Relative abundance data were analyzed by one-way ANOVA (*p < 0.05; **p < 0.01)

At the phylum level (Fig. 5a), 90% of the GM of the six groups were mainly composed of *Firmicutes* and *Bacteroides*. The abundances of *Firmicutes* in the PL, PH, PW, PE, DIC, and CON groups were 65.07, 81.13, 72.99, 76.32, 68.30, and 64.00%, respectively, while the relative abundances of *Bacteroides* were 15.13, 10.94, 15.91, 14.64, 21.05, and 18.07%. As shown in Fig. 5b, the relative abundance of *Firmicutes* was significantly higher in the PH group than the CON and PL groups (*p* ≤ 0.01). Meanwhile, the abundance of *Firmicutes* was significantly lower in the DIC group than the PH group (*p* ≤ 0.05). The relative abundance of *Bacteroides* was lower in the TCM groups than the CON group, but this difference was not significant (*p* > 0.05). *Firmicutes* is believed to help the absorb food calories and leads to weight gain, suggesting that weight gain associated with *P. oleracea* might be related to an increase in the abundance of *Firmicutes*. Also, the relative abundance of *Bacteroides* was higher in the DIC group than the CON group and gradually decreased with an increase in the dose of *P. oleracea*.

At the genus level, the GM of the six groups were mainly composed of *Lachnospiraceae, Rikenellaceae,* and *Oscillospiraceae.* As shown in Fig. 5c, the abundances of *Lachnospiraceae* in the PL, PH, PW, PE, DIC, and CON groups were 18.31, 15.49, 16.90, 17.50, 12.62, and 15.99%, respectively, while the abundances of *Rikenellaceae* were 7.26, 6.00, 8.62, 7.11, 13.17, and 11.47%. Although there was no significant difference among the six groups, DIC was found to reduce the relative abundance of *Lachnospiraceae* (*p* ≤ 0.01; Fig. 5d). However, the abundance of *Rikenellaceae* was increased in the DIC group and decreased in the other TCM treatment groups (Fig. 5d).

The F/B ratio is as an index to evaluate imbalances in the GM caused by various illnesses. As compared to the CON group, the F/B ratio was significantly increased in all of the TCM groups (*p* ≤ 0.05), but decreased in the DIC group (Fig. 5e). These results indicate that *P. oleracea* can improve the structure and maintain the stability of the GM.

LEfSe (linear discriminant analysis effect size) analysis identified significant differences in GM structure at the phylum level among the treatment groups (Fig. 5f). The results of linear discriminant analysis showed that *o__Monglobales, c__clostridia*, and *p__Fimicutes* were the dominant bacteria in the PH group, whereas *o__Oscillospiraceae, g__Monglobales*, and *g __unclassified__Veillonellales__Selenomonadales* were the dominant bacteria in the PW group. The main differentiated microbial species between the DIC and other groups were *o__Bacteroidales, f__Planococcaceac*, and *g __unclassified__Peptostreptococcaceae* (Fig. 5g). These results suggest that *P. oleracea* enhanced the proportions of beneficial bacteria, increased the F/B ratio, and restored the natural balance of the GM.

### P. oleracea increased the gut metabolite content

**Fig. 6:**
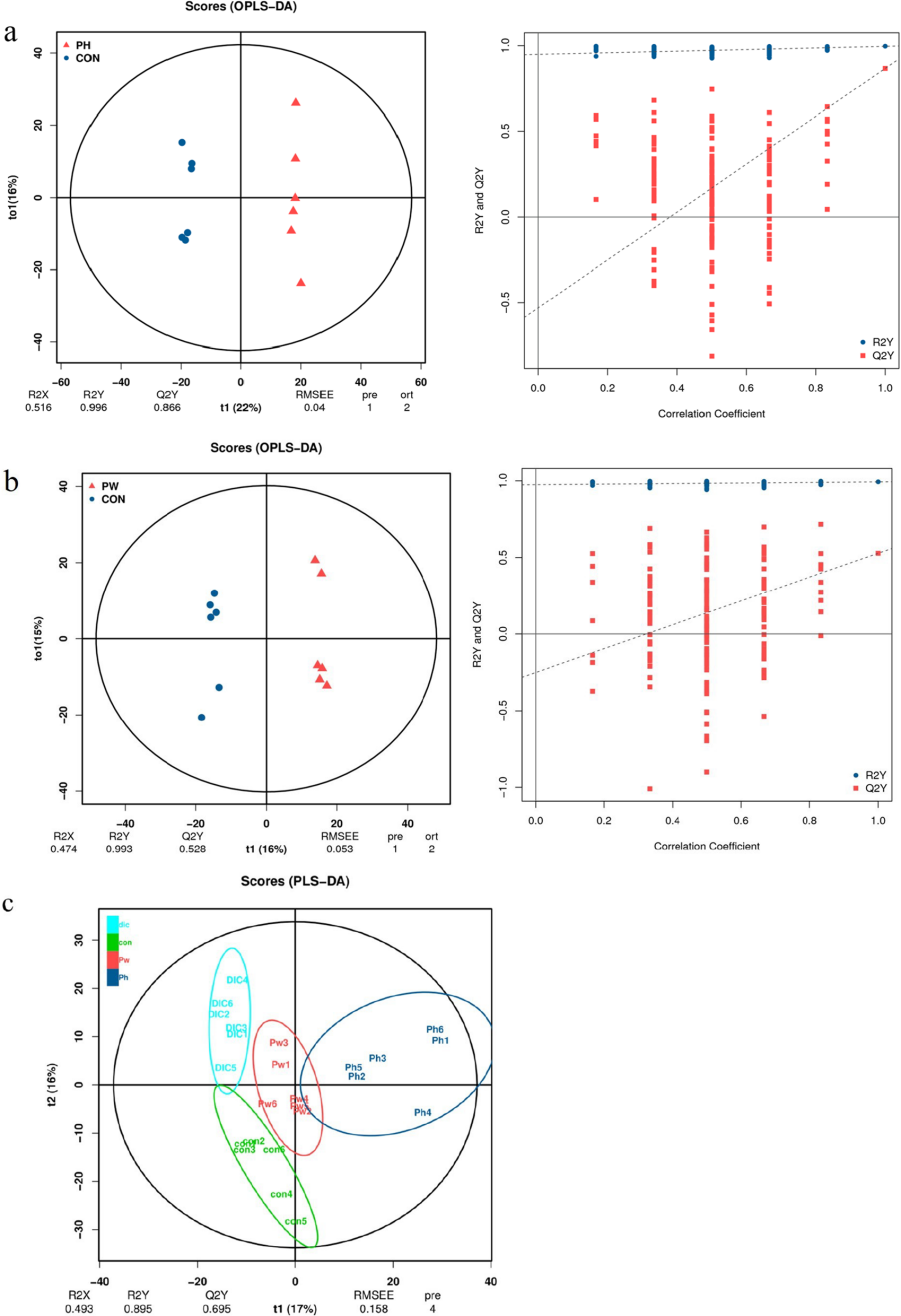

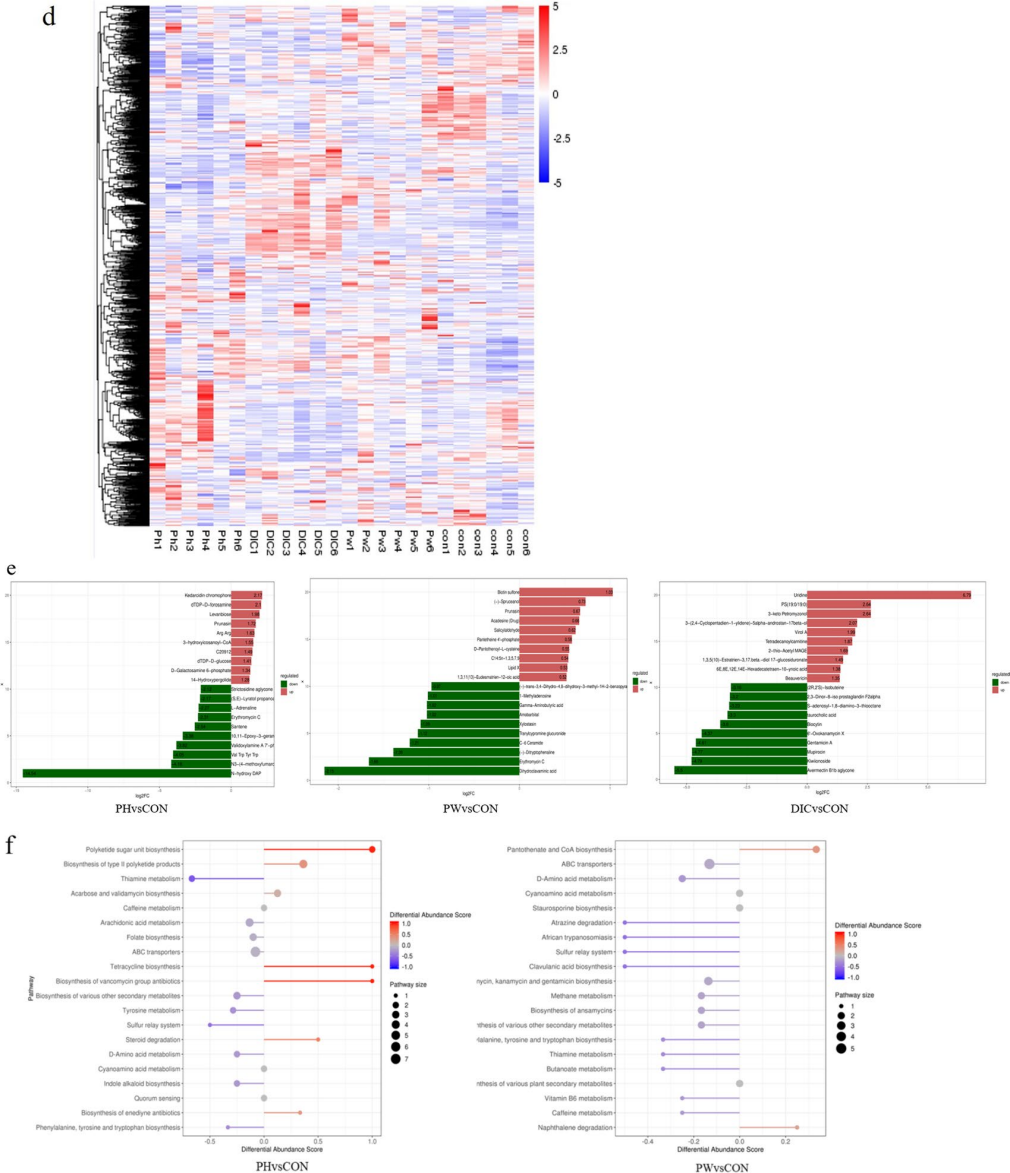

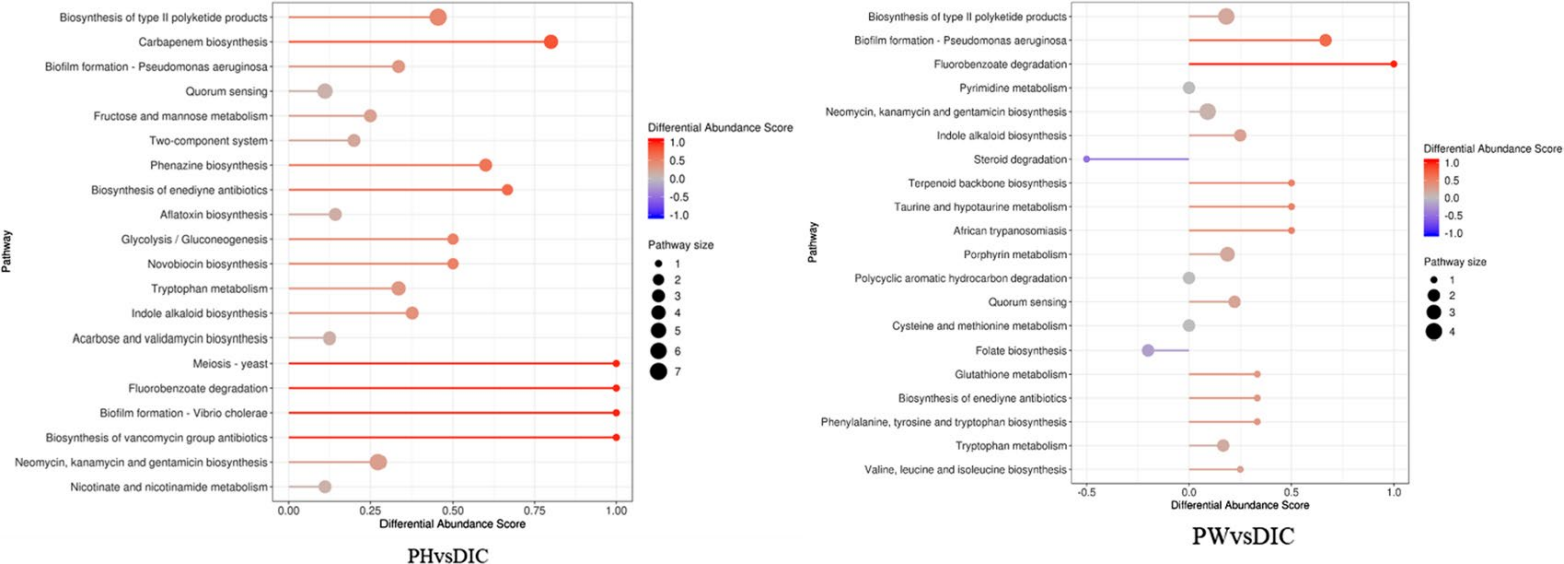
Effects on different fecal metabolites in sheep. **a** OPLS-DA score chart and permutation test (PH vs. CON). **b** OPLS-DA score chart and permutation test (PW vs. CON). The OPLS-DA score chart is often used to directly show the classification effect of the model. **c** PLS-DA of the microbial metabolites. **d** Heat map of tissue metabolites. **e** Log converted values of the differential metabolites among the groups. **f** Map of differential metabolite abundance, where the name of the differential pathway is shown on the vertical axis and the DA score is shown on the horizontal axis. The DA score reflects the overall change to all metabolites of the metabolic pathway, where a score of 1 indicates an upward trend and a score of − 1 indicates a downward trend in the expression of all annotated metabolites in the pathway

The main purpose of metabolomics analysis is to identify metabolites with significant biological activities in order to elucidate the related metabolic processes and underlying mechanisms. The results of OPLS-DA identified various differential metabolites among the treatment groups. As shown in Fig. 6a, there were significant differences in the differential metabolites between the PH and CON groups. The permutation test (R2Y = 0.996 and Q2 = 0.866) confirmed that the OPLS-DA results were reliable. There were also significant differences in metabolites between the PW and CON groups (R2Y = 0.993 and Q2 = 0.528) (Fig. 6b).

In addition, the PLS-DA results showed significant separation among the PH, PW, DIC, and CON groups (Fig. 6c), indicating the reliability of the alternative experimental model. In total, 1675 metabolites were identified and quantified in this study, which included lipids, amino acids, carbohydrates, organic acids, and esters. Cluster heat map analysis identified differences in metabolites and relative changes to metabolite concentrations among the groups. The metabolite concentrations were similar between the PW and PH groups, while there were significant differences between the DIC and CON groups (Fig. 6d).

After conducting qualitative and quantitative analyses of the detected metabolites, Fold Change values and abundance scores of the groups were compared (Fig. 6e, f). As shown in Fig. 6e, the metabolites kedaricidin chromophora, levanbiose, prunasin, and 3-hydroxycosanoyl-CoA were 2.17, 1.98, 1.72, and 1.55 times higher respectively in the PH group than the CON group. Levanbiose is reported to prevent dental caries and weight gain, and correct GM imbalance. Prunasin also has the ability to nourish the stomach and diuresis. High doses of *P. oleracea* may increase the abundance of metabolites that promote digestion, growth, and improve GM. Meanwhile, the metabolites biotin sodium, prunasin, and acadesine (drug) were 1.03, 0.67, and 0.66 times higher, respectively, in the PW group than the CON group, dihydroxyl aminic acid, gamma aminobutyric acid were 2.15, 1.02 times lower, The PW group is similar to the PH group, the content of bioactive components levambios, prunasin, and biotin sulfone are relatively increased compared to the CON group. And central nervous system inhibitory neurotransmitters that play an important role in metabolites γ- The decrease in the abundance of aminobutyric acid and acute toxic dihydroxy amino acid suggests that *purslane* may upregulate the abundance of substances that promote cell growth, fatty acid production, and fat and amino acid metabolism, thereby promoting body growth.

As shown by the map of metabolite abundance scores presented in Fig. 6f, the dots are distributed on the right side of the axis and the longer line segments indicate greater overall expression of the pathway. The differences in metabolite abundance scores between the PH and CON groups showed that the Arginine synthesis pathway, Biosynthesis of type ll polyketide products, Tetracycline biosynthesis, and Biosynthesis of vancomycin group antibiotics pathways were upregulated, while the thiamine metabolism pathway was downregulated (Fig. 6f). The metabolic pathways of Pantothenate and CoA biosynthesis and Naphthalene degradation were upregulated in the PW group, while Atrazine degradation, Clavulanic acid biosynthesis, and Sulfur relay system were down-regulated. Similar to the results of Fold Change values, the use of *P. oleracea* can up-regulate metabolic pathways such as arginine, Pantothenate and CoA that provide energy and active substances. The DIC group showed down-regulation of phenylalanine, tyrosine, and tryptophan biosynthesis; biosynthesis of enedyne antibiotics, Pseudomonas aeruginosa, Fluor benzoate degradation. In summary, *P. oleracea* has increased the abundance of certain beneficial bacteria, which promote the up-regulation of metabolic pathways of nutrients and active substances, maintain intestinal health, promote growth and development, and resist pathogens.

## Discussion

In this study, the effects of different doses of *P. oleracea*, as well as water and ethanol extracts, on fecal coccidian concentrations, BW, GM, and intestinal metabolites were investigated. The results demonstrate that *P. oleracea* reduced fecal coccidian concentrations, improved GP, maintained the composition of the GM, and reduced the F/B ratio. These findings provide a more comprehensive understanding of the therapeutic effects of *P. oleracea* against coccidiosis.

TCM is used for treatment of various diseases, including parasitic infections (Yu L et al. 2019; Liang Y et al. 2019). In the present study, low and high doses of *P. oleracea*, as well as water and ethanol extracts, ameliorated coccidiosis in sheep within 28 days in a dose-dependent manner. In the first 2 weeks, the efficacy of DIC was much higher than that of *P. oleracea*, while after two weeks, the effect of *P. oleracea* was better than that of DIC. This result is consistent with the findings of a previous study of root extract of the deciduous undershrub traditional Chinese herbal *Dichroa febrifuga* Lour (Zhang DF et al. 2012), which did not achieve complete eradication of coccidia in the early disease stage, similar to *P. oleracea*. Also, the effect of DIC had basically disappeared by short-term use and discontinuation after one developmental cycle of coccidia.

DIC is an anti-coccidian drug widely used in poultry production (El-Ashram S et al. 2019; Wang Z et al. 2020). However, due to the long-term and large-scale use of DIC, drug resistance has become problematic (Ahmadi P et al. 2022). *P. oleracea* poses a low risk of drug resistance and has shown long-lasting anti-coccidian effects, thereby demonstrating advantages as a promising treatment option. However, as compared to some herbs used in TCM with antiparasitic effects, such as Changshan, *Brucea javanica*, and *Artemisia annua*, *P. oleracea* is less effective against coccidiosis. A previous study (Lan L et al. 2016) found that as compared to hydrobromide, *B. javanica* significantly ameliorated coccidiosis, the diarrhea index, and bloody stool score of chickens. Young et al (2001) found that after 10 days of treatment with 15 herbs used in TCM for treatment of coccidiosis in chickens, *Artemisia annua* significantly reduced the lesion score and excretion of coccidian oocysts. Although the anti-coccidiocidal effect was inferior to that of chemical drugs, *P. oleracea* has the advantages of widely available sources and convenience (Kumar A et al. 2022). As compared to other non-medicinal or edible herbs that require processing into essential oils and cumbersome extraction procedures, *P. oleracea* can be directly added to feed or simply extracted with water.

In this study, dietary supplementation with *P. oleracea* effectively improved the GP of sheep. In the first two weeks, the mean DWG was significantly higher in the PE group than the DIC group, and significantly higher in PW group than the CON group. These results are consistent with the findings of a previous study that dietary supplementation with 2% and 3% *P. oleracea* significantly improved GP and increased feed conversion (Wang C et al. 2021). A prior study found that *P. oleracea* increased the GP of broilers via modulation of the GM, significantly increased the BW, improved the concentrations of immunoglobulin M and superoxide dismutase, and decreased the number of harmful bacteria (Abd El-Hack et al. 2022). The GP of sheep was found to be positively impacted by consumption of *P. oleracea*, which may be attributed to the presence of biological phenols and other active substances, reduction in parasites, and changes to the GM, or the combined actions of many factors. *P. oleracea* is widespread throughout temperate regions of China and welcome "health" edible herb in Chinese and cheap and easy to gain, it has great potential as feed additives in animal health breeding.

The roles of the GM in health and disease have been established (Kenny DJ et al. 2020). In the present study, 16S rRNA analysis was conducted to better clarify the effect of dietary *P*. *oleracea* on the GM of sheep. The results showed that the ACE and Shannon indices were higher in the PE, PW, and PH groups than the CON group, demonstrating that dietary *P*. *oleracea* improved the diversity and richness of the GM, consistent with the findings of a previous study (Yi S et al. 2022). Improving the GM can also increase absorption of dietary fibers and GP (Pascale A et al. 2019).

Meanwhile, the difference in species diversity between samples can be better illustrated through principal coordinate analysis. The closer the distance between samples on the coordinate diagram, the more similar they are. The PCoA results, based on Beta diversity analysis (Fig. 6c), indicate that the data distance matrix of the PH, PW, PE group and the CON group overlapped, while the DIC group did not overlap. The result demonstrates that the colony structure of the lambs, when treated with *P. oleracea*, is similar to the colony composition of normal lambs. This discrepancy may be attributed to the variation in the composition of TCM and Western medicine. *P. oleracea* had a positive regulatory effect on the relative abundance and diversity of the GM. Stability of the GM is necessary for the host to resist invasion of pathogenic bacteria (Wang Y et al. 2019; Liu Z et al. 2020). Hence, it is particularly important that drugs are not only effective, but also do not interfere with the stability of the GM.

*Bacteroidetes* and *Firmicutes* account for 90% of the human GM (Jandhyala SM et al. 2015). The proportions of *Firmicutes*, including *Listeria* and *Streptococcus*, are reported to decrease in response to diseases (Lanza VF et al. 2015; Wronowski MF et al. 2021). Gram-positive bacteria, such as *Lactococcus*, *Lactobacillus*, and *Listeria*, help to maintain the composition of the GM and prevent pathogen invasion (Garneau JE et al. 2008). However, *Bacteroidetes* include bacteria negatively correlated with inflammatory cytokines and are known to cause intestinal diseases (Yang X et al. 2021). In this study, the abundance of *Firmicutes* at the phylum level was significantly increased in the PH and PE groups (*p* ≤ 0.5), while the abundance of *Bacteroidetes* was decreased in the *P. oleracea* treatment groups and increased in the DIC group. These results indicate that *P. oleracea* may promote the proliferation of beneficial bacteria, but not harmful bacteria. The F/B ratio serves as an index to evaluate imbalances in the GM in response to various diseases (Liu X et al. 2020). Both *Firmicutes* and Bacteroidota play important roles in the digestion of carbohydrates and proteins, as well as maturation of the intestinal immune system. In addition, relatively high abundances of *Firmicutes* and Bacteroidota in the GM have been correlated to the energy and nutritional requirements of the host (Spence C et al. 2006). *P. oleracea* can increase the F/B ratio and improve the balance of the GM. Moreover, high levels of *Firmicutes* have been correlated to weight gain (Koliada A et al. 2017), consistent with the *P*. *oleracea* groups in the present study. At the genus level, the abundance of members of the *Lachnospiraceae* family was increased in the *P. oleracea* treatment groups and decreased in the DIC group. All members of the *Lachnospiraceae* family are anaerobic, fermentative, and chemoorganotrophic, and some exhibit strong hydrolyzing activities (Vacca M et al. 2020). *Lachnospiraceae* can produce short chain fatty acids with potential anti-inflammatory effects (Facchin S et al. 2020). The effect on GP may be related to the ability of *P. oleracea* to improve the structure of the GM and increase the abundance of beneficial bacteria, such as *Lachnospiraceae.* In this study, *P. oleracea* improved the structure of the GM, the F/B ratio, and the abundance of some probiotics, which promoted GP and digestion.

Various dietary components can induce significant changes to the composition and metabolism of the GM. In the present study, arginine content was 1.63 times higher in the PH group than the CON group, while the contents of valine and tyrosine transporters were 4.05 times lower in the CON group. Arginine is used for urea synthesis and beneficial to maintain kidney function (Morris SM. 2006). L-type amino acid transporter 1 (LAT1) is a sodium-independent transporter and mainly responsible for the transport of branched-chain amino acids, such as valine and tyrosine. Up-regulation of LAT1 has been linked to cancer progression and metastases by providing essential amino acids to tumor cells (Lu X. 2019). The contents of biotin sulfone and (-)sprucenol were higher in the PW group than the CON group. Notably, (-)sprucenol is a diterpenoid organic compound associated with cytotoxicity and anti-tumor activity (Gunasekera SP et al. 1979). Beauvericin is a naturally occurring mycotoxin that can severely damage the cell membrane, thereby allowing the release of intracellular substances (Mallebrera B et al. 2018). In the present study, the content of beauvericin was 1.35 times higher in the DIC group than the CON group, suggesting that DIC increased the content of beauvericin and is likely toxic to host cells.

The addition of *P. oleracea* can improve the structure of the GM and promote the synthesis of some bioactive metabolites. The addition of *P*. *oleracea* increased metabolism of tryptophan and nicotinic acid in the PW group as compared to the PH and DIC groups, reduced the contents of arginine and other substances, and up-regulated some vitamins and vitamin co-factors, demonstrating that TCM can impact the production of certain metabolites linked to overall health. For instance, nicotinic acid has the ability to soften blood vessels and can be used in conjunction with lipid-lowering drugs for treatment of atherosclerosis (Saggini A et al. 2011; Li T. 2015). In addition, *P. oleracea* can up-regulate production of metabolically active vitamins and certain amino acids to change the composition of the GM and improve weight gain of sheep. However, the underlying mechanisms remain unclear. The specific pathways or gut barrier effects are also unknown. Therefore, further studies are warranted to elucidate the roles of the composition of the GM in the GP and defense against parasitic diseases of sheep.

## Data Availability

All raw sequences were submitted to the NCBI Sequence Read Archive (https://www.ncbi.nlm.nih.gov/sra/) under BioProject PRJNA1061842. The data supporting the conclusion of this article are included in this article.

## Abbreviations

TCM: Traditional Chinese medicine
GP: Growth performance
GM: The gut microbiota
GP: Growth performance
OPG: The oocysts per gram
PL: Low-dose P. oleracea
PH: High-dose P. oleracea
PW: P. oleracea water ex-tract
PE: P. oleracea ethanol extract
DIC: Diclazuril
